# Modeling protein conformational transitions by a combination of coarse-grained normal mode analysis and robotics-inspired methods

**DOI:** 10.1186/1472-6807-13-S1-S2

**Published:** 2013-11-08

**Authors:** Ibrahim Al-Bluwi, Marc Vaisset, Thierry Siméon, Juan Cortés

**Affiliations:** 1 CNRS, LAAS, 7 avenue du colonel Roche, F-31400 Toulouse, France; 2 Univ de Toulouse, LAAS, F-31400 Toulouse, France

## Abstract

**Background:**

Obtaining atomic-scale information about large-amplitude conformational transitions in proteins is a challenging problem for both experimental and computational methods. Such information is, however, important for understanding the mechanisms of interaction of many proteins.

**Methods:**

This paper presents a computationally efficient approach, combining methods originating from robotics and computational biophysics, to model protein conformational transitions. The ability of normal mode analysis to predict directions of collective, large-amplitude motions is applied to bias the conformational exploration performed by a motion planning algorithm. To reduce the dimension of the problem, normal modes are computed for a coarse-grained elastic network model built on short fragments of three residues. Nevertheless, the validity of intermediate conformations is checked using the all-atom model, which is accurately reconstructed from the coarse-grained one using closed-form inverse kinematics.

**Results:**

Tests on a set of ten proteins demonstrate the ability of the method to model conformational transitions of proteins within a few hours of computing time on a single processor. These results also show that the computing time scales linearly with the protein size, independently of the protein topology. Further experiments on adenylate kinase show that main features of the transition between the open and closed conformations of this protein are well captured in the computed path.

**Conclusions:**

The proposed method enables the simulation of large-amplitude conformational transitions in proteins using very few computational resources. The resulting paths are a first approximation that can directly provide important information on the molecular mechanisms involved in the conformational transition. This approximation can be subsequently refined and analyzed using state-of-the-art energy models and molecular modeling methods.

## Background

Conformational transitions in proteins are generally related to their capacity to interact with other molecules. Their study is therefore essential for the understanding of protein functions. Unfortunately, it is very difficult to obtain this type of dynamic information at the atomic scale using experimental techniques. Modeling protein conformational transitions with conventional computational methods is also challenging because, in many cases, these transitions are rare, slow events. Standard molecular dynamics (MD) simulations with current computational resources cannot be applied in practice to model large-amplitude (slow time-scale) conformational transitions. Such simulations require variants of MD methods that enhance sampling of rare events or that bias the exploration in a given direction (e.g. [1-5]), or, alternatively, to have access to outstanding computational power [6].

Modeling conformational transitions in proteins has motivated the development of specific methods, computationally more efficient than MD simulations. Many of these methods (e.g. [7-9]) are based on the deformation of a trivial initial path between the two given conformations toward the minimum energy path connecting them. Consequently, the performance of these methods is strongly conditioned by the suitability of the initial path. In recent years, methods to model conformational transitions have also been developed on the basis of robot motion planning algorithms [10-13]. Most of these robotics-inspired methods are aimed at providing qualitative information about the conformational transition using few computational resources. For this, they exploit the efficiency of sampling-based exploration algorithms applied to simplified molecular models.

The high dimensionality of the space to be explored is the main difficulty that all computational methods to model protein conformational transitions have to face. Therefore, several approaches have been developed to reduce the dimensionality of the problem (e.g. [14-16]). Normal mode analysis (NMA) [17] is a particularly interesting tool in this regard, since a small number of low-frequency normal modes provide a good hint of the direction of large-amplitude conformational changes [18-21]. Several recent works apply this property of NMA to improve the performance of conformational exploration methods.

The approach presented in this paper was originally introduced in [22]. The basic principle is to use NMA to bias the conformational exploration performed by a Rapidly-exploring Random Tree (RRT) algorithm [23], aiming to efficiently compute conformational transition paths. The main novelty presented in the present work is the introduction of a multi-scale model for the protein. In this model, an elastic network is defined considering only a single node (called a particle) per tripeptide. Motion directions provided by NMA of such a coarse-grained elastic network are then applied to the all-atom model for a more accurate conformational exploration. The introduction of this multi-scale model has important outcomes. First, the number of normal modes is largely reduced thanks to the use of the coarse-grained model, which significantly reduces the time required to compute them. In addition, generating the all-atom model from the coarse-grained model can be accurately and efficiently achieved using methods from robot kinematics [24], avoiding the need of artifacts such as the RTB approach (rotations-translations of blocks) [25].

Next section presents the overall method, and explains each of its elementary components: elastic network normal mode analysis, tripeptide-based multi-scale protein modeling, and motion-planning-based conformational exploration. Then, several types of results aimed to validate the approach and to show its good computational performance are presented for a set of proteins with different sizes and topologies. A more detailed analysis of results is presented for adenylate kinase (ADK). Finally, together with the conclusions, we discuss possible directions for future work. Note that a preliminary version of this work was presented in [26]. Compared to this previous version, this paper includes more detailed explanations of the method, a more exhaustive presentation of results, with additional figures and tables, as well as additional results for the ADK protein. In addition, some movies that illustrate results obtained with the proposed method are included as supplementary material.

## Methods

This section presents a new method to model protein conformation transitions. It builds on the combination of several components inside an iterative algorithm. One of these components is NMA performed on a coarse-grained elastic network model of the protein, which enables very fast computation of normal modes. Indeed, a single particle of the elastic network is considered for each group of three consecutive amino-acid residues (i.e. one particle per tripeptide). The all-atom model, which is used to accept or reject sampled states during the conformational exploration, is accurately reconstructed from the coarse-grained one using closed-form inverse kinematics. The RRT algorithm is applied to explore linear combinations of normal modes computed from intermediate conformations along the path. All these elementary components of the method are further explained below.

### Elastic networks and normal mode analysis

Based on a harmonic approximation of the potential energy, normal mode analysis provides information about the directions and frequencies of vibration of a molecule from a minimum-energy conformation. Each mode represents a motion pattern, in which all the atoms move with the same frequency and phase. Low-frequency normal modes correspond to collective motions (e.g. domain motions), whereas high-frequency normal modes correspond to local fluctuations [19,27].

Normal modes are calculated by diagonalizing the Hessian matrix of the potential energy of the molecule. For reducing the computational cost of this operation, several works propose to use simplified potentials and coarse-grained models. An extensively used simplified potential is based on the elastic network model (ENM) [28], which represents the molecule as a set of particles connected by virtual springs. All the protein atoms can be considered as particles in the elastic network. However, a coarse-grained representation that only considers C*_α _*atoms (i.e. a single particle per amino-acid residue) is often applied [19,20]. Moreover, particles are connected by virtual springs only if they are closer than a user-defined cut-off distance *d_cut_*.

The potential energy function of such an elastic network takes the following form:

$$E=\underset{d_{ij}^{0}<d_{cut}}{\sum }\frac{C}{2}{\left(d_{ij}-d_{ij}^{0}\right)}^{2}$$

where *d_ij _*is the distance between particle *i *and particle *j*, $d_{ij}^{0}$ is the distance between the two particles at the equilibrium state and *C *is the elastic constant. This type of simplified potential has been used in many works and for very different applications [29-32].

**Figure 1:**
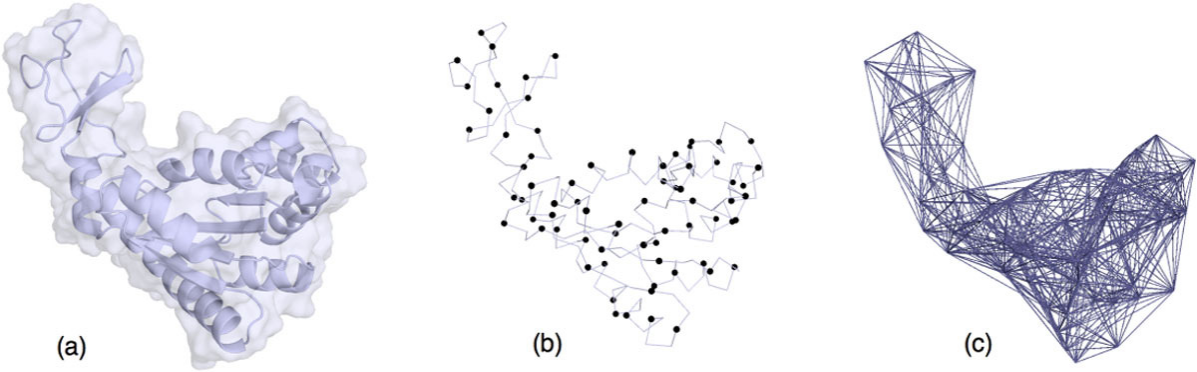
**Illustration of the different models on the ADK protein**. (a) Representation of the all-atom model, (b) the particles of the coarse-grained tripeptide-based model, (c) representation of the elastic network model.

In this work, we investigate a further simplification of the ENM. Indeed, the ENM is built using a coarser model based on tripeptides, instead of using C*_α _*atoms. Figure 1 illustrates the approach. Note that coarse-grained NMA approaches considering more than one residue per particle have already been proposed [25,33,34]. However, these approaches, which are mainly devised to analyze motions of very large systems made of protein assemblies, consider rigid-body motions of groups of residues. In contrast, the approach presented here preserves full flexibility of the protein, which leads to a more accurate simulation of conformational transitions.

Several works show that using a simplified ENM does not necessarily imply a loss of accuracy in the prediction of large-amplitude motion directions [20,25]. However, it certainly leads to a computational performance gain. This issue is further discussed in the results section, where the performance of NMA using tripeptide-based models and C*α*-based models is compared.

The anisotropic network model (ANM) approach, as described in [27,35], is adopted in this work to construct the Hessian matrix from the positions of the particles of the tripeptide-based model. Each 3 *× *3 sub-matrix corresponding to the interaction between two particles is computed as follows:

$$H_{ij}=-\frac{C}{d_{ij}^{2}}\left[\begin{array}{ccc} \left(x_{j}-x_{i}\right)\left(x_{j}-x_{i}\right) & \left(x_{j}-x_{i}\right)\left(y_{j}-y_{i}\right) & \left(x_{j}-x_{i}\right)\left(z_{j}-z_{i}\right)\\ \left(y_{j}-y_{i}\right)\left(x_{j}-x_{i}\right) & \left(y_{j}-y_{i}\right)\left(y_{j}-y_{i}\right) & \left(y_{j}-y_{i}\right)\left(z_{j}-z_{i}\right)\\ \left(z_{j}-z_{i}\right)\left(x_{j}-x_{i}\right) & \left(z_{j}-z_{i}\right)\left(y_{j}-y_{i}\right) & \left(z_{j}-z_{i}\right)\left(z_{j}-z_{i}\right) \end{array}\right]$$

$$H_{ij}=-\underset{j|j\neq i}{\sum }H_{ij}$$

If the distance between particles *i *and *j *is more than the cut-off distance *d_cut_*, then the whole 3 *× *3 matrix is replaced by zeros. The Hessian matrix is then diagonalized to compute the eigenvalues and eigenvectors. Each eigenvalue and eigenvector pair corresponds to one normal mode, where the eigenvalue defines the mode frequency and the eigenvector defines the motion direction for each particle in the elastic network.

### Multi-scale model

#### Tripeptide-based model

The multi-scale modeling approach applied in this work is based on a decomposition of the protein chain into fragments of three amino acid residues, which we refer to as *tripeptides*. The reason for choosing such a subdivision is that, assuming fixed bond lengths, bond angles and peptide bond torsions, the backbone of a tripeptide involves 6 degrees of freedom (three pairs of angles *φ, ψ*), and thus, an analogy can be made with a 6*R *mechanism like a robotic manipulator [24]. Two Cartesian reference frames attached to the N atom in the backbone of the first residue and to the C atom in the last residue define respectively the *base-frame *and the *end-frame *of the 6*R *mechanism. Since tripeptides are linked through rigid peptide bonds, the location of the end-frame of tripeptide *i *can be determined from the base-frame of tripeptide *i *+ 1 by a constant 3D transformation. Given the location of the base-frame and the end-frame, the conformation of a tripeptide backbone can be obtained by *inverse kinematics*. Consequently, the conformation of the whole protein backbone can be determined from the pose of a single reference frame attached to each tripeptide (this is true for all the protein backbone except two short fragments at the N-terminal and C-terminal ends of the chain, which require a particular treatment). In the following, we will refer to these reference frames as (oriented) *particles*. They are the particles in the coarse-grained ENM.

#### Reconstructing the all-atom model

The interest of the decomposition of the protein into tripeptides explained above is that closed-form inverse kinematics (IK) can be applied to reconstruct the all-atom protein model from the coordinates of the particles. The IK solver applied in this work has been adapted from the method developed by Renaud [36]. This solver is based on algebraic elimination theory, and develops an ad-hoc resultant formulation inspired by the work of Lie and Liang [37]. Starting from a system of equations representing the IK problem, the elimination procedure leads to an 8-by-8 quadratic polynomial matrix in one variable. The problem can then be treated as a generalized eigenvalue problem, as proposed in [38], for which efficient and robust methods such as the Schur factorization can be applied. Note however that our approach is not dependent on this solver, so that other IK methods (e.g. [38,39]) could be applied.

In general, the IK problem for a 6R serial kinematic chain has a finite number of solutions (up to 16 in the most general case). All the solutions correspond to geometrically valid conformations of the tripeptide backbone with fixed ends defined by the pose of the particles. However, when the goal is to simulate continuous motions, the closest conformation to the previous one (i.e. the one before a perturbation applied to the particles) has to be selected in order to avoid jumps in the conformational space. All IK solutions are rejected if none of them remains within a distance threshold that depends on the perturbation step-size.

The explanations above concern only the reconstruction of the all-atom model of the protein backbone from the coarse-grained tripeptide-based model. Side-chains are treated separately, using a simple method based on energy minimization as explained below.

### Path finding algorithm

The path finding method works by iteratively generating short portions of the transition between two given conformations of a protein, which we will refer to as *q_init _*and *q_goal_*. Algorithm 1 presents the pseudo-code with the main steps of the method. At each iteration, normal modes are computed for a root conformation *q_root_*. Note that *q_root _= q_init _*for the first iteration. Then, the RRT algorithm is applied to explore motions corresponding to linear combinations of normal modes. RRT is run until the protein moves a predefined distance toward the target conformation *q_goal_*. The conformational exploration performed by the RRT algorithm is further explained below. Once the RRT exploration is stopped, the closest node *q_close _*in the tree to *q_goal _*is searched. The path between *q_root _*and *q_close _*is then extracted and saved. All the conformations in this path are guaranteed to have a collision-free backbone (including C*_β _*atoms) which generally implies getting acceptable energy values after a short minimization to rearrange side-chain conformations. Such an energy minimization procedure is performed on *q_close_*, which will be the root conformation in the next iteration. The algorithm keeps iterating until a predefined distance *d_target_*to *q_goal _*is reached. The resulting path is defined by the sequence of minimized conformations *q_close _*at each iteration. If a finer-grained path is required, other intermediate conformation can be extracted from the sub-paths computed at each iteration. These conformations may require energy minimization to rearrange side-chains, as it is done for *q_close_*.

**Algorithm 1: **COMPUTE_PATHWAY

**input **: Initial conformation *q_init_*, final conformation *q_goal_*, minimum distance to target *d_target_*

**output **: The transition path *p*

begin

    *q_root _← q_init_*;

    **while **RMSD(*q_root_, q_goal_*) >*d_target _***do**

        *a ← *COMPUTE_NORMAL_MODES(*q_root_*);

        *t ← *BUILD_RRT(*q_root_, q_goal_, a*);

        *q_close _← *CLOSEST_TO_TARGET(*t, q_goal_*);

        *q_root _*← MINIMIZE(*q_close_*);

        *p ← *CONCATENATE(*p, q_root_*);

end

**Algorithm 2:** BUILD_RRT

**input **: Initial conformation *q_root_*, final conformation *q_goal_*, normal modes *a*

**output **: The tree *t*

begin

    *t ← *INIT_TREE(*q_root_*);

    **while not **STOP_CONDITION(*t, q_goal_*) **do**

        *q_rand _← *SAMPLE(t, a);

        *q_near _← *BEST_NEIGHBOR(*t, q_rand_*);

        *q_new _← *EXPAND_TREE(*q_near_, q_rand_*);

        **if **ISVALID*(q_new_) ***then**

            ADD_NEW_NODE(*t, q_new_*);

            ADD_NEW_EDGE(*t, q_near_, q_new_*);

end

### Implementation details

The RRT algorithm, iteratively applied in Algorithm 1, performs the same steps as the basic RRT [23]. The steps are sketched in Algorithm 2. At each iteration, a conformation *q_rand _*is randomly sampled. Note that *q_rand _*is not required to be a feasible conformation. Then, the tree is searched for the closest conformation to *q_rand_*, called *q_near _*. A new conformation, *q_new _*, is generated by moving from *q_near _*towards *q_rand _*with a predefined short step size. The new conformation is added to the tree if it does not violate feasibility constraints, which in the present work are limited to geometric constrains related to no atom overlapping and no bond breaking. The difference with respect to the basic RRT algorithm concerns the implementation of the methods for sampling conformations, searching the nearest neighbor, and expanding the tree. These methods, which are further explained below, are specific to the present framework because of the multi-scale protein model and the application of NMA to bias the exploration.

#### Sampling random conformations

The idea is to randomly sample conformations *q_rand _*using information given by the normal modes. The coarse-grained tripeptide-based model is used at this level. Hence, *q_rand _*is not an all-atom conformation, but an array of particle positions. Random particle positions are generated by moving them from their initial positions, defined by *q_root_*, using a linear combination of normal modes with randomly sampled weights. More precisely:

- A sequence of 3*n *random weights *w_j _*are sampled in the range [-1, 1], where *n *is the number of particles, being 3*n *the number of normal modes (actually, the number of normal modes is 3*n − *6, since 6 degrees of freedom correspond to rigid-body motions of the whole set of particles).

- The new positions of the *n *particles are computed by a linear combination of all the randomly weighted modes as follows:

$$q_{rand}=q_{root}+\overset{3n}{\underset{}{\sum }}f*w_{j}*a_{j}$$

where *a_j _*refers to each normal mode, and *f *is an amplification factor used to push the sampled conformation away from *q_root _*(this factor is the same for all the normal modes). Note that, since the normal modes are not normalized, low frequency modes have larger norm. Thus, they contribute more significantly in the sum.

#### Finding nearest neighbors

Nearest neighbor search is also performed using the coarse-grained model. Indeed, the computed distance is based on the root mean squared deviation (RMSD) of the particle positions. In the current implementation, the distance is biased to pull the exploration towards the target conformation as follows:

$$d\left(q,q_{rand}\right)=\text{RMSD}\left(q,q_{rand}\right)\frac{\text{RMSD}\left(q,q_{goal}\right)}{\text{RMSD}\left(q_{init},q_{goal}\right)}.$$

In this work, we have implemented a simple brute-force algorithm to find *q_near_*. More sophisticated nearest neighbor search algorithms could be used to reduce the number of performed distance computations. Note, however, that currently used algorithms based on space partitioning techniques (e.g. kd-trees) do not perform well in high-dimensional spaces [40]. A computationally efficient solution would require the implementation of an approximate nearest neighbor search algorithm.

#### Generating new conformations

For generating *q_new_*, all particle positions in *q_near _*are linearly interpolated towards *q_rand _*with a predefined step size *k*. Given these new particle positions, the all-atom model corresponding to *q_new _*is obtained by solving an IK problem for every tripeptide. The implemented method proceeds iteratively. If no IK solution is found for a tripeptide *t_i_*(the tripeptide between particles *p_i _*and *p*_*i*+1_) or if the solution involves atom collisions, the pose (position and orientation) of particle *p*_*i*+1 _is slightly perturbed and the IK problem is solved again. This process is repeated until a collision-free IK solution is found or a maximum number of trials is reached. If this process fails to find a collision-free IK solution for any tripeptide, failure is reported and the RRT algorithm goes back to the random sampling step.

Once the treatment of all tripeptides has been completed, the conformation of the two terminal fragments is generated. For this, the pose of these fragments is updated with respect to the new poses of the first and last tripeptides. Random perturbations can be applied to these end fragments in order to remove possible collisions with the rest of the protein.

Protein conformations *q_new _*generated using the aforementioned process are guaranteed to satisfy geometric constraints: correct bond geometry and no overlap betweew backbone atoms. However, in order to speed-up computations, side-chains are not treated at this stage (only C*_β _*atoms are considered for collision avoidance). This is because side-chains are known to be very flexible, and resolving possible collisions along the conformational transition path can be done in a post-processing stage. Indeed, side-chain collisions are resolved during the minimization step at the end of each short RRT execution.

## Results and discussion

This section discusses several experiments aimed to validate the proposed method and to evaluate its performance. First, the question concerning the accuracy of the tripeptide-based elastic network model is addressed. Then, results are presented on conformational transitions computed for a set of ten proteins with different sizes and topologies. Finally, further results on adenylate kinase are presented and compared to available data on the transition between the open and closed forms of this protein.

### Validating the coarse-grained ENM

Previous works (e.g. [19,20]) have shown that simple ENMs built using C*_α _*atoms perform as well as ENMs built using the all-atom model when studying the dynamic properties of proteins with NMA. Here, we compare the performance of the proposed tripeptide-based model with the C*_α_*-based model for predicting directions of conformational transitions. A set of seven proteins listed in Table 1 was used for this comparison. These proteins were also used in related work [20] for the validation of the C*_α_*-based ENM.

**Table 1:** Proteins used in the overlap experiments

Protein	Residues	PDB_*open*_	PDB_*closed*_
Che Y Protein	128	3chy	1chn
LAO binding Protein	238	2lao	1laf
Triglyceride Lipase	256	3tgl	4tgl
Thymidulate Synthase	264	3tms	2tsc
Maltodextrine Binding Protein	370	1omp	1anf
Enolase	436	3enl	7enl
Diphtheria Toxin	523	1ddt	1mdt

For evaluating the capability of normal modes to predict directions of conformational transitions, we use the notion of *overlap *as proposed in related work [20]. The overlap *I_j _*between a normal mode *j *and an experimentally observed conformational change between two conformations (open and closed) *q^o^*and *q^c^*is defined as a measure of similarity between the conformational change and the direction given by the normal mode *j*. It can be computed as follows:

$$I_{j}=\frac{\left|\overset{3n}{\underset{}{\sum }}a_{ij}\text{Δ}q_{i}\right|}{{\left[\overset{3n}{\underset{}{\sum }}a_{ij}^{2}\overset{3n}{\underset{}{\sum }}\text{Δ}q_{i}^{2}\right]}^{1/2}}$$

where $\text{Δ}q_{i}=q_{i}^{o}-q_{i}^{c}$ measures the difference between the particle coordinates in conformations *q^o^*and *q^c^, a_ij _*corresponds to the *i^th ^*coordinate of the normal mode *j*, and *n *is the number of particles. A value of 1 for the overlap means that the direction given by the normal mode matches exactly the conformational change, whereas a value around 0.2 or less means that the normal mode is unable to provide any meaningful prediction.

**Figure 2:**
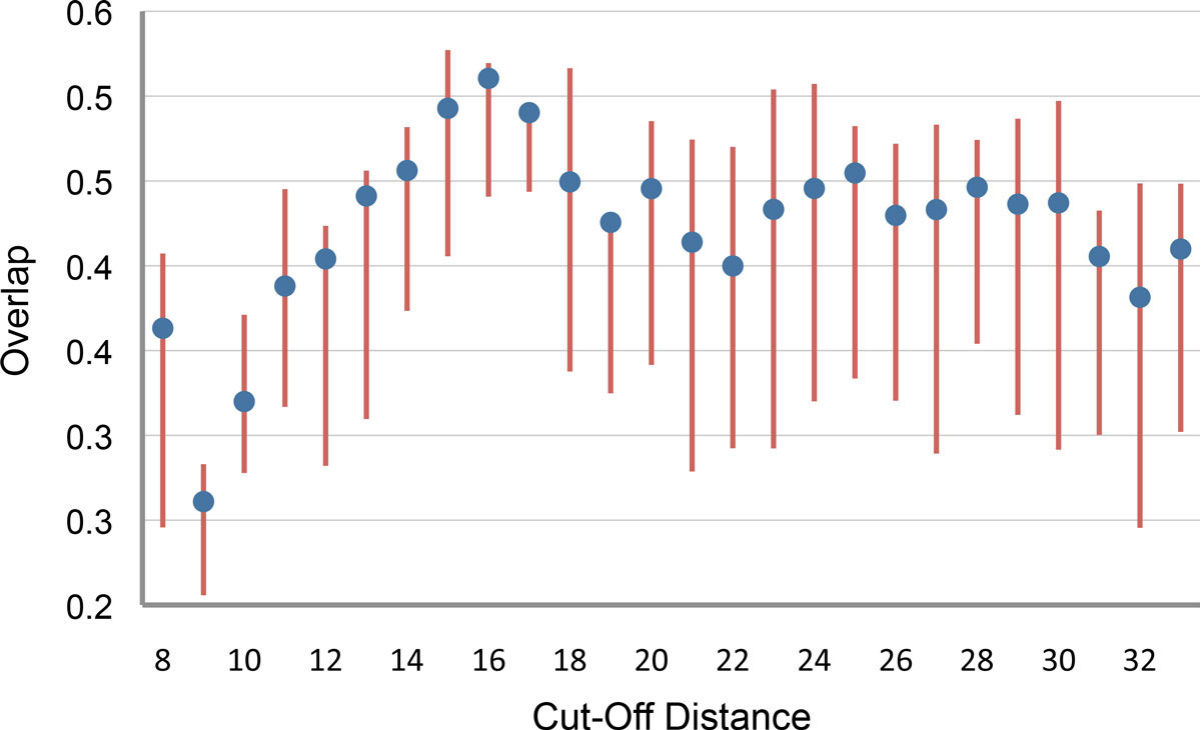
**Average overlap over the seven proteins of Table 1**. Lines are drawn between the 25th and the 75th percentiles of the overlap values. Average overlap values are indicated with dots.

Before conducting the comparative analysis, we need to determine an optimal cutoff distance for the tripeptide-based ENM. A good cutoff distance should create an elastic network that correctly captures the topology of the protein. For C*_α_*-based models, 8 Å is generally used, since this cutoff distance has been empirically shown to provide the best results in most cases. It can be intuitively inferred that the same cutoff distance may not be the optimal choice in our case, because distances between particles of the tripeptide-based model are larger than distances between C*_α _*atoms. Moreover, defining the optimal cutoff value theoretically is not straightforward. Therefore, we have measured and compared the overlap values for the seven proteins with cutoff distances between 8 and 34 Å in order to empirically determine the most suitable range of cutoff values. Figure 2 shows the overlap value for each cutoff distance averaged over the seven proteins. Note that, for each protein, overlap values were computed for all the normal modes, and the best value was considered for the average. As clearly shown in the figure, the best overlap values are for cutoff distances of 15, 16 and 17 Å.

**Figure 3:**
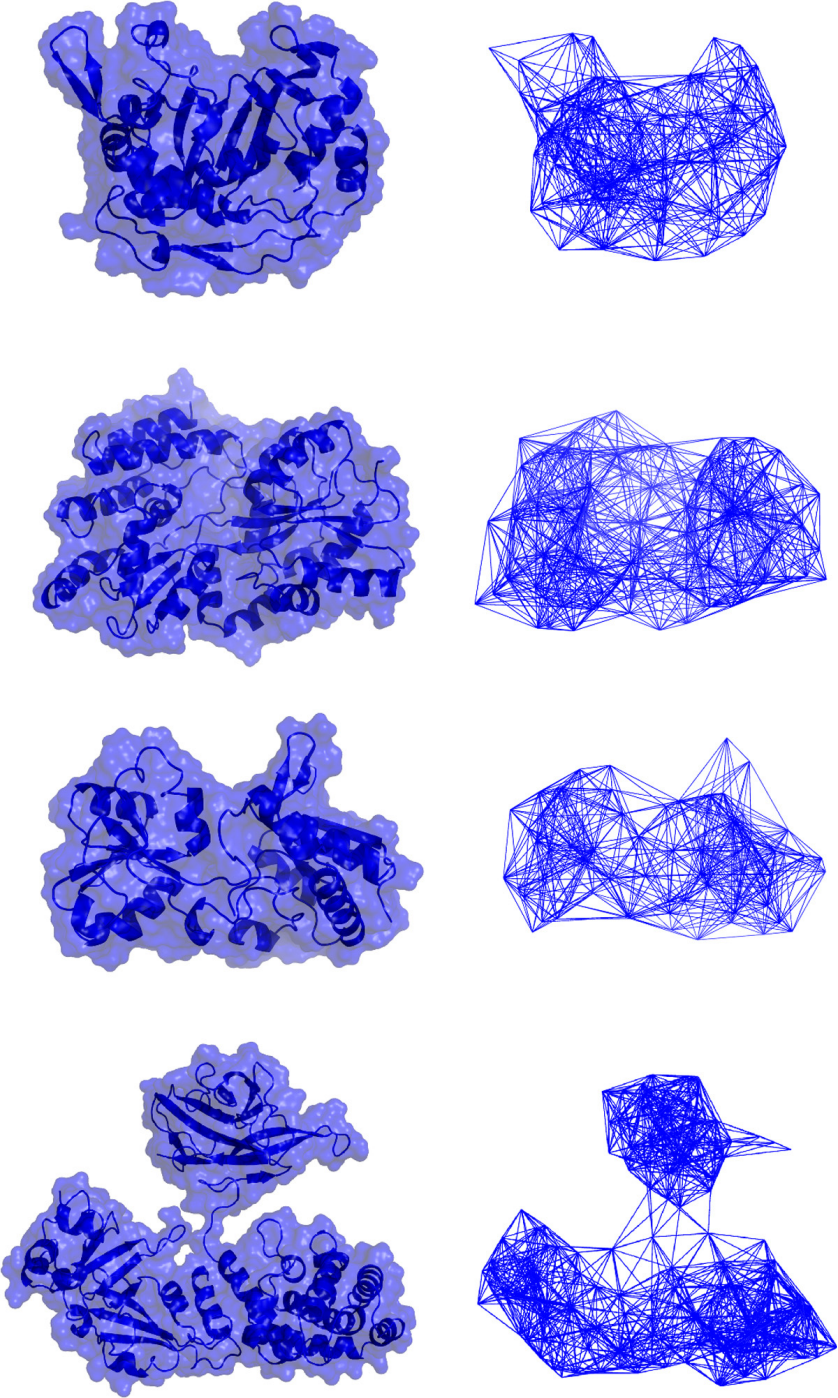
**Tripeptide-based elastic network models**. Representation of the all-atom models and the tripeptide-based ENMs for four different proteins.

The tripeptide-based ENMs for four of the proteins in Table 1, using a cutoff distance of 16 Å, are represented in Figure 3. The figure shows that the main topological features of the proteins appear in the coarse-grained model.

Table 2 compares overlap values of tripeptide-based ENMs using a cutoff distance of 16 Å with those presented in [20] for C*_α_*-based ENM using a cutoff distance of 8 Å. In the table, columns labeled "Open" correspond to the open-to-closed conformation and columns labeled "Closed" are for the opposite case. The similar overlap values show that the coarse-garined, tripeptide-based ENM is also able to capture the topological information required to compute normal modes that correctly predict directions of large-amplitude motions. Importantly, such a similar performance in terms of overlap is obtained with much less computational cost. Since the computational complexity of the Hessian matrix diagonalization is $O\left(n^{3}\right)$, the reduction of *n *by a factor 3 (a tripeptide involves 3 C*_α _*atoms) provides a theoretical gain of more than one order of magnitude. This theoretical gain has been confirmed with some experiments. In summary, the time required to compute the normal modes with the tripetide-based model ranges from 0.05 seconds to 0.9 seconds, whereas several minutes may be necessary using the C*_α _*model.

**Table 2:** Comparison between overlap values for C_α_-based ENMs and tripeptide-based ENMs

Protein	C_*α *_Overlap		Tripep. Overlap	
		
	Open	Close	Open	Close
Che Y Protein	0.32	0.34	0.52	0.34
LAO binding Protein	0.84	0.40	0.53	0.52
Triglyceride Lipase	0.30	0.17	0.26	0.35
Thymidulate Synthase	0.56	0.40	0.49	0.29
Maltodextrine Binding Protein	0.86	0.77	0.90	0.84
Enolase	0.33	0.30	0.40	0.30
Diphtheria Toxin	0.58	0.37	0.48	0.30

### Finding conformational transitions

#### Experimental setup

**Figure 4:**
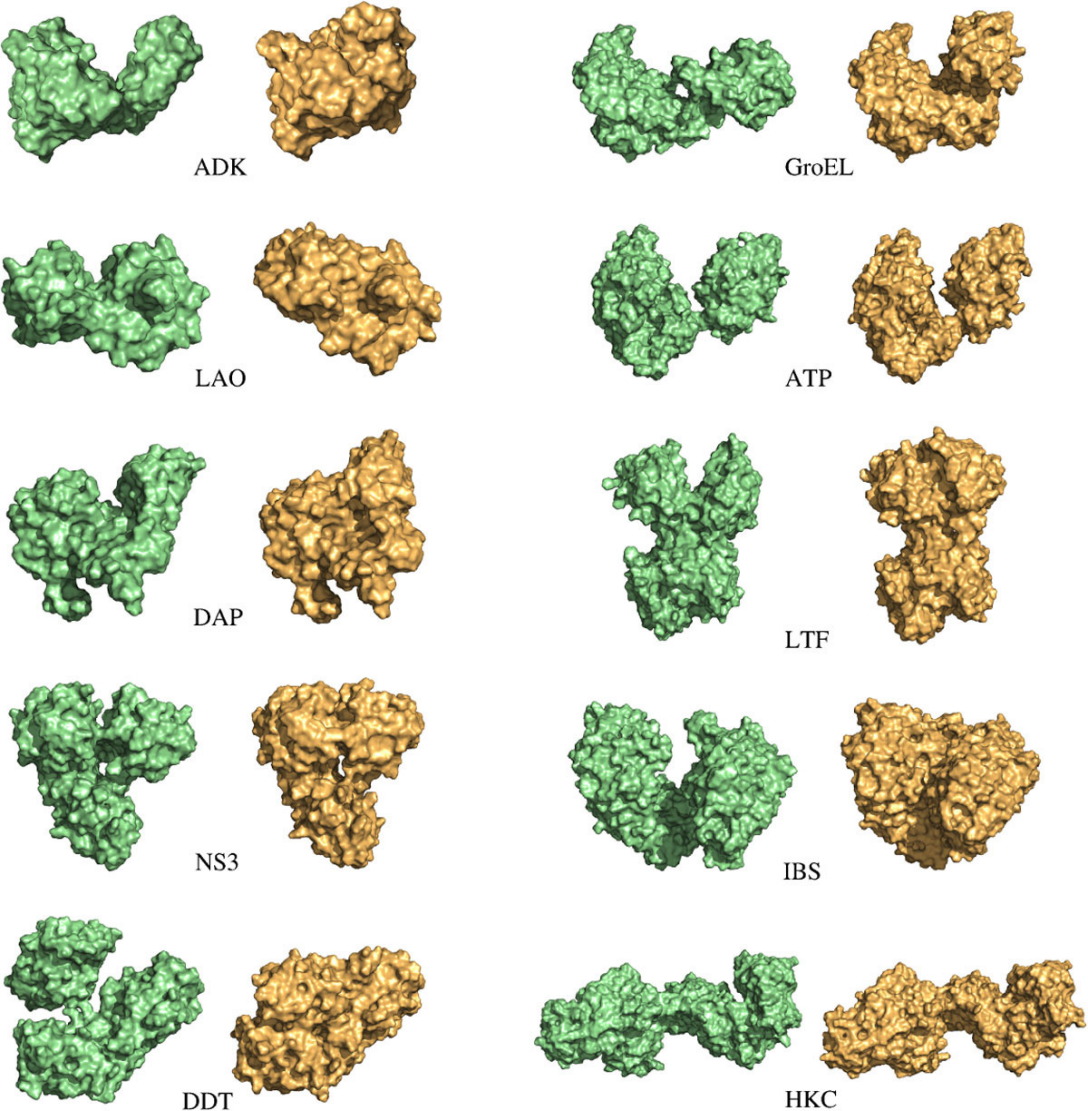
**The ten proteins used in the experiment**. Representation of open and closed forms of these proteins available in the PDB (IDs are provided in Table 3).

The proposed method was applied to compute conformational transition paths for the ten proteins listed in Table 3, and represented in Figure 4. For each protein, at least two experimental structures corresponding to different conformations are available in the Protein Data Bank (PDB) [41]. The difference between these conformations involves large-amplitude domain motions. The ten proteins are varied in size and topology, as well as in the type of domain motions they undergo. This heterogeneity is important to analyze the reliability and scalability of the method.

**Table 3:** Proteins used in the experiments

Protein	Residues	PDB ID_*init*_	PDB ID_*goal*_	C_*α *_RMSD
ADK	214	4ake	1ake	6.51
LAO	238	2lao	1laf	3.73
DAP	320	1dap	3dap	3.78
NS3	436	3kqk	3kql	2.75
DDT	535	1ddt	1mdt	10.96
GroEL	547	1aon	1oel	10.49
ATP	573	1m8p	1i2d	3.78
LTF	691	1cb6	1bka	4.75
IBS	876	1ukl	1qgk	6.17
HKC	917	1hkc	1hkb	3.00

Each iteration of the algorithm that computes the transition path performs a short RRT exploration, as mentioned in the previous section. In the current implementation, such a local exploration runs until the protein moves 0.3 Å C*_α_*-RMSD towards the goal. This distance is gradually reduced to 0.15 Å as the distance to the target conformation decreases. The reason is that the speed of convergence tends to decrease when approaching the target conformation, and recomputing normal modes more frequently provides better results in this situation. If the distance stopping condition is not reached first, the exploration stops after a pre-defined number of iterations (4000 in our case). This additional stopping condition prevents too long runs of RRT in case of blocking situations.

At the end of the RRT exploration, the closest conformation to the goal is identified and submitted to an energy minimization procedure aimed at generating better side-chain conformations. In this work, we have used the AMBER software package [42] for energy minimization.

#### Results

Table 4 summarizes the results achieved by the proposed method for the set of ten proteins. In this table, C*α*-RMSD*_end _*is the distance between the goal conformation and the conformation obtained at the end of the iterative path finding process. Time*_total _*is overall computing time, which includes the RRT running time (Time*RRT *) and the time for computing the normal modes and running minimizations at the end of each iteration. The number of iterations of the main algorithm (i.e. the number of NMA calculations) is also indicated in the table. Note that, in all the experiments, the RRT exploration takes more than 90% of the total computing time, which corresponds to runs on a single core of an AMD Opteron 148 processor at 2.6 GHz.

**Table 4:** Performance of the method on ten proteins (cf. Table 3)

Protein	C_*α*_-RMSD_*end*_	Iterations	Time_*RRT*_	Time*_total_*
ADK	1.56	31	1.82	2.00
LAO	1.32	20	1.52	1.65
DAP	1.31	16	1.78	1.92
NS3	1.29	14	2.82	3.00
DDT	2.88	272	81.54	86.4
GroEL	2.79	142	40.21	42.17
ATP	1.45	30	13.46	14.16
LTF	1.96	74	29.56	31.09
IBS	1.99	80	80.61	82.62
HKC	1.64	38	37.91	39.63

**Figure 5:**
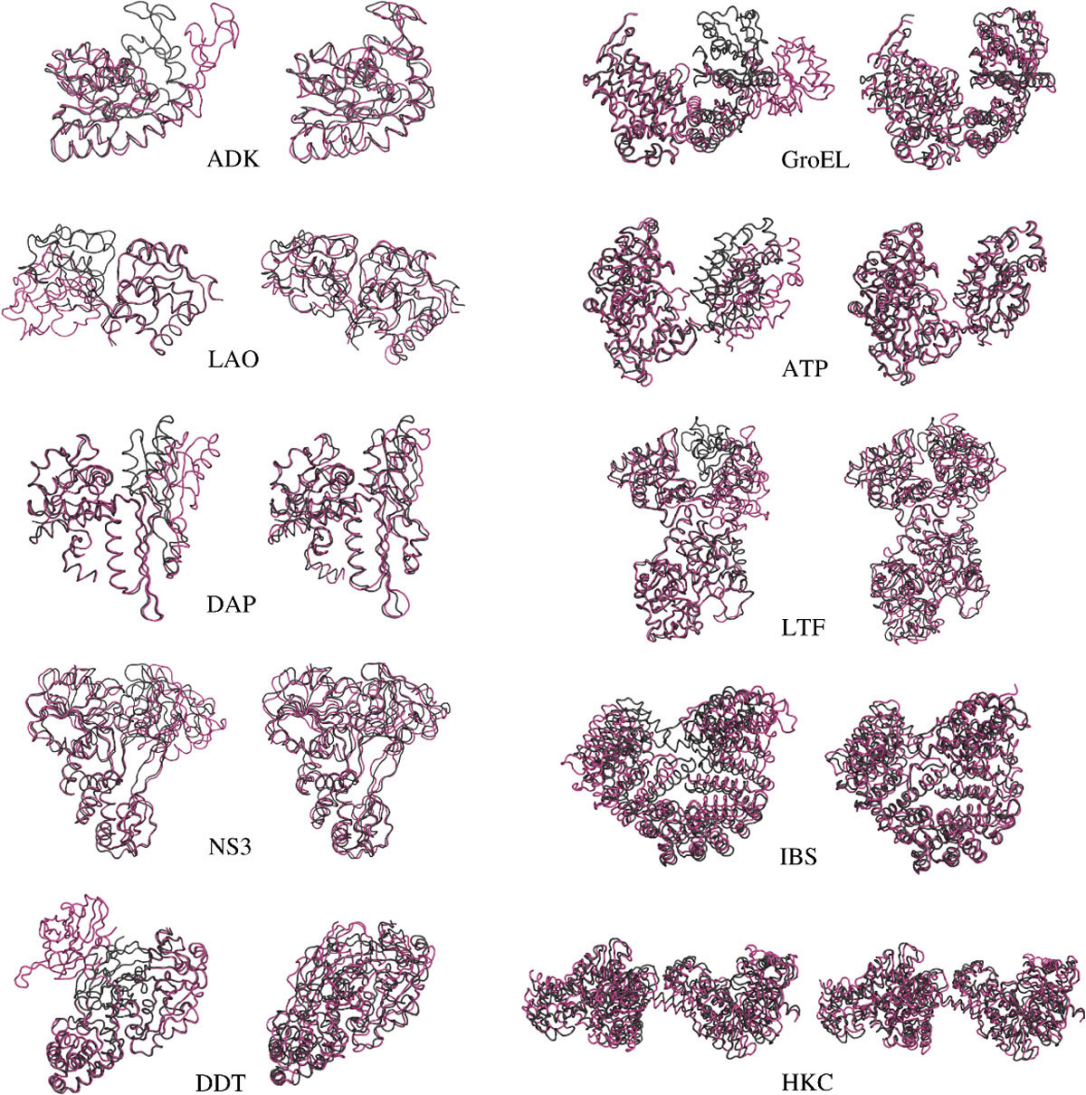
**Superimposed structures and final conformations of the computed transition path**. For each protein, the left image shows the open form (in red) and the closed form (in black), and the right image shows the closed form (in black) and the final conformations of the computed path (in red).

In all cases, the method was able to compute the conformational transition, reaching conformations very close to the given goal conformations. Figure 5 shows superimposed structures (structure superimpositions and images have been done using PyMOL [43]) of open and closed forms of the proteins (*q_init _*and *q_goal_*), and of the closed form and the last conformation of the computed transition path (*q_goal _*and *q_final_*). The distances between the final and goal conformations are below 2 Å (measured using C*_α_*-RMSD) for all the tested proteins with the exception of DDT and GroEL. Note that 2 Å RMSD corresponds to the current accuracy of experimental methods for high-resolution protein structure determination. As can be seen in Figure 5 the superimpositions of the final and goal conformations is very good, even for DDT and GroEL. Note that the method could have reached closer conformations to the goal with a higher number of iterations. Nevertheless, the strategy applied in these experiments was to stop iterating when the distance to the goal reached a very slow rate of convergence.

**Figure 6:**
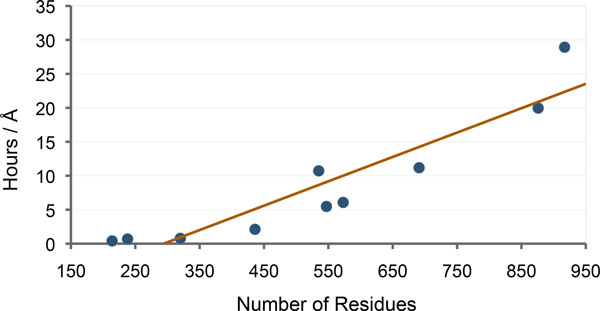
**Plot of the results in Table 5**. The plot shows a linear relationship between the size of the protein and the time required to compute the conformational transition path.

We also conducted experiments to analyze the relationship between the computing time and the size of the protein. Since the lengths of the transition paths for the different test systems is variable, we measured the computing time to move 1Å along these paths. The results of these experiments, presented in Table 5 and Figure 6, show a linear relationship between the computing time and the protein size. This scalability is an interesting property of the method. Note that the performance of the method seems not be (or only slightly) affected by the topology of the protein. This is an important advantage over the method presented in [22], which experienced some difficulties in dealing with relative motions of domains connected by several linkers, mainly because of the internal-coordinate representation of proteins used in this previous work.

**Table 5:** Relationship between the size of the protein and the computing time

Protein	Residues	Time (hours)
ADK	214	0.4
LAO	238	0.68
DAP	320	0.79
NS3	436	2.11
DDT	535	10.72
GroEL	547	5.84
ATP	573	6.74
LTF	691	11.17
IBS	876	19.96
HKC	917	28.93

Finally, we did a profiling of the algorithm to identify possible bottlenecks and points to be improved to enhance computational efficiency. Table 6 gives values of the percentage of the time spent in the most time-consuming operations within the RRT exploration: nearest neighbor search (NN), collision checking (CC), inverse kinematics (IK) and random sampling (RS). Surprisingly, nearest neighbor search takes around 60% of the overall computing time. This is due to the brute-force algorithm applied in the current implementation. As mentioned before, a more sophisticated nearest neighbor algorithm should be implemented. The performance of the method could also be enhanced by applying simplified distance metrics (e.g. [16,44]). The use of an appropriate simplified distance metric could reduce computing time while preserving good exploration properties of the algorithm.

**Table 6:** Percentage of the time spent performing the main operations in RRT

Protein	NN	CC	IK	RS
ADK	57.2%	14.1%	15.0%	6.3%
LAO	51.3%	20.9%	17.0%	5.4%
DAP	50.5%	20.6%	11.0%	12.3%
NS3	67.9%	13.4%	6.6%	8.9%
DDT	64.3%	17.1%	6.9%	9.0%
GroEL	60.4%	17.6%	8.9%	9.8%
ATP	57.3%	20.9%	6.8%	11.9%
LTF	55.1%	16.8%	6.1%	19.3%
IBS	62.9%	15.5%	4.1%	15.5%
HKC	68.9%	5.8%	3.3%	18.2%

Average	59.58%	16.27%	8.57%	11.66%

#### A closer look at adenylate kinase

Adenylate kinase (ADK) [45] is a widely studied protein involved in signal transduction. The structure of ADK is composed of three domains known as: LID, CORE and NMPbind. Several works tend to show that the LID and NMPbind domains undergo large-amplitude conformational changes with respect to the CORE domain, which remains stable [46,47]. Some of these works (e.g. [47]) also suggest that the conformational transition between open and closed states of ADK proceeds in two steps: (1) the LID domain moves more clearly than the NMPbind domain at the beginning of the open-to-close transition; (2) then NMPbind domain moves at a faster pace towards the end of the transition path.

**Figure 7:**
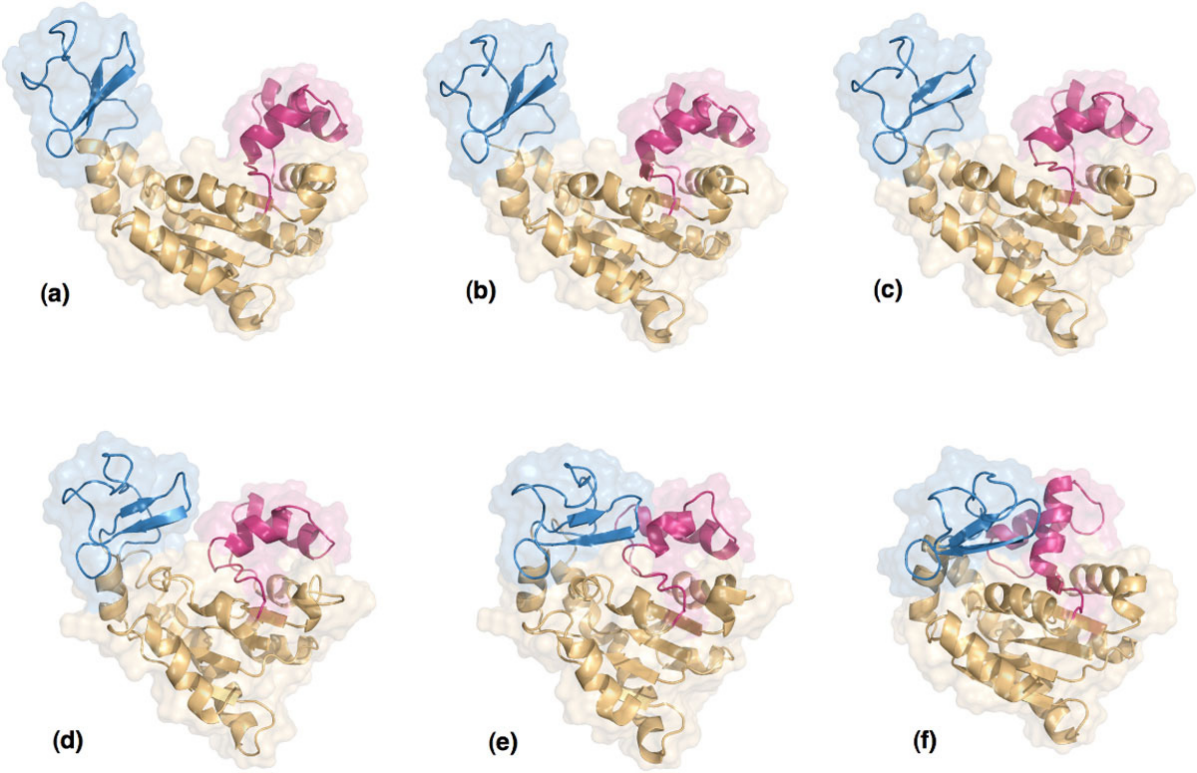
**Different conformations of ADK along the studied conformational transition**. The LID domain is shown in blue and the NMPbind domain is shown in red. Images (a) and (f) represent the start and goal conformations respectively. Images (b) to (e) show intermediate conformations generated by our method.

**Figure 8:**
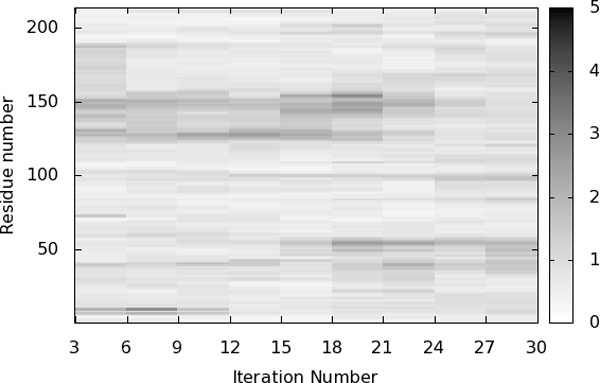
**Displacement of the residues along the conformational transition of ADK**. The plot shows, using a gray-scale, the displacement of each residue at each iteration relative to the previous iterations. Darker regions represent larger displacements.

The open conformation of ADK (PDB ID 4AKE), the closed conformations (PDB ID 1AKE) of ADK, and several intermediate conformations obtained with our method are represented in Figure 7 The figure shows significant conformational changes of the LID and NMPbind domains, as expected. The motion of these two regions is also illustrated in Figure 8, which represents the displacement of the residues along the conformational transition. Two darker regions, involving residues 20-60 and 130-160, indicate the parts of the protein that undergo larger displacements. These regions correspond to the NMPbind domain and LID domain, approximately. Figure 8 also shows that residues 20-60, corresponding to the NMPbind domain, start moving more significantly near the end of the transition path, whereas residues 130-160, corresponding to the LID domain, start moving at an earlier stage. This reflects the two-step nature of the conformational transition discussed earlier, and shows that our method provides results that are qualitatively comparable with those presented in previous work on ADK.

The open conformation of ADK (PDB IDs 4AKE), the closed conformations (PDB IDs 1AKE) of ADK, and several intermediate conformations obtained with our method are represented in Figure 7 The figure shows significant conformational changes of the LID and NMPbind domains, as expected. The motion of these two regions is also illustrated in Figure 8, which represents the displacement of the residues along the conformational transition. Two darker regions, involving residues 20-60 and 130-160, indicate the parts of the protein that undergo larger displacements. These regions correspond to the NMPbind domain and LID domain, approximately. Figure 8 also shows that residues 20-60, corresponding to the NMPbind domain, start moving more significantly near the end of the transition path, whereas residues 130-160, corresponding to the LID domain, start moving at an earlier stage. This reflects the two-step nature of the conformational transition discussed earlier, and shows that our method provides results that are qualitatively comparable with those presented in previous work on ADK.

We have also compared intermediate conformations in the computed transition path of the ADK to a small number of other experimentally solved structures of this protein. These structures correspond to homolog proteins or mutants with very high sequence identity, and some of them are known to be intermediate structures between open and closed forms of the protein. Interestingly, four of these structures are very close to conformations along the transition path. Table 7 shows the distance between each of these structures and the closest conformation in the transition path. The table also shows the position of this conformation in the path. More precisely, the table shows the corresponding iteration number and the percentage of the path length. 2RH5 (A) is very close to the conformation generated by the first iteration, whereas 1E4Y (A) is close to the conformation generated by iteration 27 (near the closed structure). 1DVR (A) is also very close to a conformation toward the beginning of the path (near the open structure), whereas 2RH5 (B) is a slightly less open structure. These results are comparable to those provided by previous studies [12,48], which further validates the proposed method.

**Table 7:** Known intermediate structures and their distances to the closest conformation in the computed transition path.

PDB ID	RMSD	Iteration	Path percent
1DVR (A)	1.48	2	9%
2RH5 (A)	1.80	1	4%
2RH5 (B)	1.91	3	15%
1E4Y (A)	2.20	27	94%

## Conclusions

This paper has presented an efficient approach for computing large-amplitude conformational transitions in proteins. It exploits the ability of normal modes to predict directions of collective, large-amplitude motions and the efficiency of the RRT algorithm to explore large spaces. The proposed approach also relies on a multi-scale representation of the protein, based on a decomposition into tripeptides, which significantly contributes to the good performance of the method.

Interestingly, first results presented in the paper show that using an ENM based on the coarse-grained tripeptide-based model instead of a C*α*-based model preserves the ability of NMA to predict directions of large-amplitude motions, while significantly reducing computing time.

The proposed method was applied to simulate large-amplitude conformational transitions in proteins of different sizes and topologies. Results show a good performance of the method in all the cases. Computing time scales linearly with the number of residues. It ranges from a few hours for medium-size proteins to a few days for very large ones. This computational performance could be significantly improved by the implementation of more sophisticated methods to perform the most time-consuming operations within the RRT algorithm, in particular, nearest neighbor search.

A deeper analysis of the conformational transition between open and closed forms of ADK shows that results provided by the proposed method are qualitatively consistent with results obtained with other computational methods and with experimental data. Nevertheless, it is important to note that the resulting paths are a first approximation, which cannot be used directly for an accurate evaluation of energy variations along conformational transitions. This would require a subsequent refinement and analysis using state-of-the-art energy models and molecular modeling methods. It could also be possible to integrate energy evaluations within the RRT exploration with the aim of obtaining better-quality solutions, at the expense of additional computational cost. An interesting extension that could be investigated is to use T-RRT [49,50], instead of RRT, to compute paths that follow more accurately the valleys of the conformational energy landscape.

In this work, we have shown the ability of the proposed method to compute transition paths between two given conformations of a protein. Nevertheless, the approach could also be applied to more challenging problems, such as the prediction of other (meta-)stable states reachable from a given protein conformation, or the discrimination between probable and improbable transitions. This would require some extensions, mainly in the definition of energy/scoring functions to identify interesting intermediate and meta-stable states, as well as high-energy barriers, during the conformational exploration.

## List of abbreviations

MD: molecular dynamics; NMA: normal mode analysis; RRT: rapidly-exploring random tree; RTB: rotations-translations of blocks; ENM: elastic network model; ANM: anisotropic network model; IK: inverse kinematics; RMSD: root mean squared deviation; PDB: Protein Data Bank.

## Competing interests

The authors declare that there are no competing interests.

## Authors' contributions

TS and JC designed this research and supervised the work. IA implemented the method and carried out experiments. MV participated in the software design and implementation. IA and JC wrote the manuscript. All authors have read and approved the manuscript.

## Additional files

## Supplementary Material

Additional file 1 Movie of a conformational transition path for AKD Click here for file

Additional file 2 Movie of a conformational transition path for GroEL Click here for file

Additional file 3 Movie of a conformational transition path for LTF Click here for file

Additional file 4 Movie of a conformational transition path for IBS Click here for file

## Declarations

The publication costs for this article were funded by the French National Research Agency (ANR) under project ProtiCAD (project number ANR-12-MONU-0015-01).

This article has been published as part of *BMC Structural Biology *Volume 13 Supplement 1, 2013: Selected articles from the Computational Structural Bioinformatics Workshop 2012. The full contents of the supplement are available online at http://www.biomedcentral.com/bmcstructbiol/supplements/13/S1.
